# A New Filling

**Published:** 1872-03

**Authors:** 


### EDITORIAL DEPARTMENT.

---

*A New Filling*.—At the April meeting of the "Odontological Society of Great Britain," Mr. Fletcher described his new method of filling teeth as follows:

"The filling is a mixture of two parts of silica ground to an impalpable powder, and one of gum copal. It requires to be broken into pieces which are small enough to go into the cavity, and afterwards to be condensed with a hot smooth instrument, which is kept slightly moistened with oil to prevent adhesion. It can be built up to any form, and afterwards filed to shape, the edges carefully condensed with a warm instrument, and the surface polished. The easiest way to keep the instrument slightly oily is to have at hand a gun-wad, or small piece of felt soaked in oil, on which it can be occasionally rubbed. It is in some cases better to soften the pieces of filling over a flame before inserting them in the cavity; but they must be inserted and condensed very quickly, as they become hard as stone in two or three seconds, if not kept hot. This sudden hardening is one difficulty which it requires practice to overcome. I have used the filling for about three months in all positions, and the only case in which I have had the slightest change in it, is in the first filling I made with it, which was not properly condensed. It is not intended to replace gold, and I did not wish any one to suppose so; but it is, so far as I have seen, a good cement, and more trustworthy than any other we have, approaching the color of the teeth. The copal wears slowly off the surface of the compound, leaving a surface of silica as smooth as glass, which appears unchangeable. I shall be glad to send a small quantity to any one who will try it carefully and report on it. If more is wanted, the process of making has been published, and no doubt the depots will take the matter up."

